# Supplementary material for: Ultrafast-nonlinear ultraviolet pulse modulation in an AlInGaN polariton waveguide operating up to room temperature
Source: Nat Commun. 2021 Jun 9;12:3504. doi: 10.1038/s41467-021-23635-6 (PMC8190124; doi:10.1038/s41467-021-23635-6)
Supplement: Supplementary file 1 — Supplementary Information [file 41467_2021_23635_MOESM1_ESM.pdf]

Supplementary material for ultrafast-nonlinear ultraviolet pulse modulation in an AlInGaN polariton waveguide operating up to room temperature

D. M. Di Paola,[1] P. M. Walker,[1,*] R. P. A. Emmanuele,[1] A. V. Yulin,[2] J. Ciers,[3,4] Z. Zaidi,[1] J.-F. Carlin,[3] N. Grandjean,[3] I. Shelykh,[2,5] M. S. Skolnick,[1,2] R. Butté,[3,†] and D. N. Krizhanovskii[1,2]

[1]*Department of Physics and Astronomy, University of Sheffield, S3 7RH, Sheffield, UK*
[2]*Department of Physics, ITMO University, St Petersburg, 197101, Russia*
[3]*Institute of Physics, École Polytechnique Fédérale de Lausanne (EPFL), CH-1015 Lausanne, Switzerland*
[4]*Department of Microtechnology and Nanoscience, Chalmers University of Technology, 412 96 Gothenburg, Sweden*
[5]*Science Institute, University of Iceland, Dunhagi-3, IS-107 Reykjavik, Iceland*

Supplementary Discussion 1. Sample Details

The sample we investigate is nominally identical to the one described in supplementary reference 1. The active region consists of $N = 22$ GaN QW layers of thickness 1.5 nm sandwiched between 3.5 nm-thick $Al_{0.1}Ga_{0.9}N$ barriers. The active region is grown by metal-organic vapor-phase epitaxy and sandwiched between a 400 nm-thick $Al_{0.83}In_{0.17}N$ bottom cladding that is lattice matched to the GaN substrate, and a 175 nm-thick SiO_2 top cladding. The gratings for light input and output were realized with a 20 nm-thick nickel metallisation beneath the SiO_2 cladding. They have a 130 nm periodicity with 65 nm metal stripes and were fabricated using electron-beam lithography, thermal evaporation of the metal and lift-off.

The photonic properties of the waveguide modes in the absence of strong photon-exciton coupling were calculated using the CAMFR package for the Python programming language[2], freely available from `http://camfr.sourceforge.net`. Following the procedure used in supplementary references 1 and 3, where the linear properties of these AlInGaN polariton waveguides are already extensively described, we obtained the AlInGaN refractive indexes from the formulae given in supplementary references 4 and 5. The refractive indexes are plotted as a function of wavelength in Supplementary Figs. **1**a-b for two temperatures. We note that the refractive index formulas are only strictly valid for frequencies below the material band edge, which results in the discontinuity visible in the refractive index for the GaN buffer layer. However, since the lower polariton branch is by definition at frequencies below the quantum well exciton energy, and since the guided modes are isolated from the GaN buffer layer by evanescent decay in the the AlInN cladding, this does not present a problem for our calculations. Supplementary Figure **1**c shows the calculated waveguide photonic mode dispersions for several temperatures. The modes curve rather than being straight because of the frequency dependent refractive index of the materials. Supplementary Figure **1**d shows the guided mode electric field intensity as a function of position along the growth direction z of the waveguide, along with the corresponding refractive index $n(z)$. This confirms that the guided mode is well localised in the waveguide core containing the quantum wells and decays rapidly in the AlInN cladding.

Supplementary Discussion 2. Photoluminescence measurements

Figure 2a in the main text shows the exciton spectra for several sample temperatures. Excitation at 325nm was performed using 20 nano-Joule femotsecond laser pulses apart from the curve labelled '4K CW', for which a 75 micro-Watt CW HeCd laser was used. By comparing this with the curve labelled '4K' it can be seen that there is no significant difference in the PL between the CW and pulsed excitation despite the very different peak power levels. The spectral features agree well with those already reported for the nominally identical sample in supplementary reference 1. At 4 K the highest energy peak corresponds to the quantum well A exciton X_A and a bound exciton peak is visible on its low energy side. Emission from the GaN buffer layer can be seen as a large peak $\sim$ 130 meV lower in energy than X_A. Fitting the X_A peak at 4 K gives a full width at half maximum of 9.6 ± 0.3 meV. These observations confirm that our sample indeed has the same electronic properties as supplementary reference 1.

Figure 2b in the main text shows the PL emitted into polariton states after it has propagated to the grating couplers and been scattered out. This PL was excited using the CW HeCd laser at 325nm. Excitation power was 2.5 mW in all cases apart from the curve labelled '4 K 0.25 mW'. It can be seen that for all temperatures there are broad PL peaks at lower energy than the exciton. At 4 K and 100 K the peaks are close to 35 meV below X_A, denoted by one of the vertical dashed black lines. There are also secondary peaks close to 92 meV below X_A corresponding the the first LO phonon replica of the exciton. These observations are again consistent with supplementary reference 1. For increasing temperatures the peak near -35 meV reduces in intensity compared to that near -92 meV. This may be due to increased propagation losses for polaritons nearer to X_A. We especially note that excitation power has little effect on the shape of the PL spectrum, as can be seen by comparing the two curves at 4 K which were excited with an order of mag-

nitude difference in power. The peak 35 meV below X_A is ~ 30 meV wide and the total spectrum is ~ 75 meV wide including the LO phonon replica. This is expected since the hot carrier relaxation incoherently populates all available lower energy states so that the spectrum is broad even at low powers and stays broad with increasing power. We further note that the incoherent nature of PL precludes nonlinear broadening mechanisms.

Supplementary Discussion 3. Dispersion measurement and fitting

To measure the polariton waveguide frequency (wavelength) vs. wavenumber (angle) dispersion relation we performed angle-resolved photo-luminescence measurements. It is important to note that these measurements are quite different to the resonant pulse transmission experiments which are the main subject of the manuscript, but they play an important role by confirming that the sample is in the strong photon-exciton coupling regime from 4 K to 300 K.

We excited our structure with a CW HeCd laser emitting at $\lambda_0 = 325$ nm, above the quantum well barrier band gap, at various points between two grating couplers set 200 µm apart. The incident laser spot for these non-resonant measurements was elliptical with full width at half maximum (FWHM) 7 µm x 11 µm. Hot-carrier relaxation populates all polariton states, which propagate to the diffractive grating couplers and are scattered out at angles near normal to the sample surface (the normal corresponds to 0° in Figs. 2c,d in the main text). The Fourier plane of the microscope objective used to collect the light was imaged onto the entrance slit of an imaging spectrometer using a pair of relay lenses and recorded on a thermo-electrically cooled CCD camera. For these dispersion measurements the spectral resolution was limited to 6.7 meV (FWHM) by the large (200 µm) entrance slit size of the spectrometer used to obtain a good signal to noise ratio owing to the low intensity of the photoluminescence. Projecting the Fourier plane of the emission onto an entrance slit of this size corresponds in our case to averaging over a transverse angular range (angle in the y direction) of $\sim \pm 1.1°$ outside the sample or $\theta_{\mathrm{T}} \sim \pm 0.4°$ inside the waveguide (by Snell's law, using the guided mode effective index). This is equivalent to a frequency change $\omega \sin^2 \theta_{\mathrm{T}}$ of only 0.2 meV and so the averaging over transverse angles has a negligible effect on resolution. The relation between CCD pixel number and emission angle was carefully calibrated by placing a commercial 300 lines/mm UV diffraction grating in the position of the sample and recording the diffraction pattern. Luminescence spectra from the sample were resolved in polarisation using a broadband UV half wave plate and wire grid linear polariser. The spectra cross-polarised with the TE-polarised polariton mode were subtracted from the co-polarised spectra to remove the exciton luminescence while retaining the polariton mode emission.

In Figs. 2c,d in the main text two copies (LPB_1 and LPB_2) of the lower polariton branch (LPB) dispersion relation can be seen, symmetric about zero angle, which are due to polaritons propagating in the forward and backward x directions in the waveguide. The white dashed curves give the exciton wavelength (X) and the uncoupled waveguide photon dispersion (Γ) calculated from an electromagnetic model of the planar waveguide eigenmodes (see Supplementary Discussion 1). The experimental angle vs. wavelength dependence of the LPB was extracted from the luminescence spectra and fit by a coupled oscillator model. We were able to extract the central wavelength of the emission peak for each angle with low uncertainty (the exact value of uncertainty depends on the emission strength at the angle in question but is typically less than ± 0.5 meV), much lower than the spectrometer resolution, by fitting the peaks with Gaussian functions. We extracted between 44 and 85 wavelength vs. angle points for the spectra taken at different temperatures.

To fit the extracted wavelength vs. angle dispersion we used a coupled oscillator model with three variable parameters - the exciton frequency E_{X}, the Rabi splitting Ω (which gives the photon-exciton coupling rate), and a rigid frequency offset E_Γ of the uncoupled photon mode Γ compared to the raw uncoupled photon mode dispersion obtained from the eigenmode calculation. The last parameter accounts for the fact that there are inevitably small differences between the nominal and actual layer thicknesses and/or temperature dependent refractive indexes. The solid white curves in Figs. 2c,d show the LPB obtained from the fit. There is good agreement between the model and the luminescence spectra, demonstrating that the system is strongly coupled up to room temperature. We note that including E_Γ as a fitting parameter was essential to minimise the square error of the fit r^2. Without it we obtained smaller values for the Rabi splitting but much worse fits, e.g. 3.5 times the r^2 error when fitting 44 points with 2 parameters rather than 3. The additional uncertainty in Ω and the exciton frequency due to including E_Γ as a fitting parameter is included in the quoted values. The values of E_Γ obtained from the fit are less than 15 meV or 0.5% of the photon frequency, consistent with small errors in the material parameters entering into the transfer matrix model. For this reason we believe our Rabi splitting values ~ 90 meV to be reliable even though they are larger than those found in supplementary reference 1 which were effectively fit using the raw photonic modes from finite-difference time-domain (FDTD) simulations.

Finally, we discuss the slight asymmetry between LPB_1 and LPB_2 in Fig. 2c in the main text. For this data the laser excitation spot overlapped the edge of one of the gratings. LPB_1 corresponds to the polariton mode that propagates away from the excitation onto the main body of the grating and couples out. By contrast, the emission from LPB_2 can only come from the section of grating directly under the excitation spot since the spot is on the

edge of the grating and polaritons propagating away in the LPB_2 direction simply enter the un-grated region and are never detected. LPB_2 thus presents a nonlinear frequency blue shift of up to ~ 7 meV (0.7 nm) compared to LPB_1 and to the modelled LPB dispersion, which arises from the relatively high exciton, polariton and potentially also free carrier densities generated directly under the spot. We note that we measured the dispersion for a number of spot positions relative to the gratings and confirmed that the spectra are symmetrical when the excitation spot is between the two gratings.

Supplementary Discussion 4. Resonant excitation and detection of propagating polariton pulses

In order to study the pulse propagation through the waveguide, we excited our structure through a grating coupler with laser pulses of the same frequency and wavevector as the polariton mode (see Fig. 1 in the main text). Contrary to the dispersion curve measurements, in this case we *directly* inject particles into the polaritonic waveguide guided mode without carrier relaxation. We inject pulses with a range of central frequencies (and corresponding angles) through an input grating coupler and detect the output from a second grating placed at a distance $L = 100\,\mu$m. The UV laser pulses were generated by frequency-quadrupling the output of a tuneable optical parametric amplifier pumped by the 800 nm pulses from a Ti:sapphire regenerative amplifier. To match the bandwidth of the sample grating couplers the initially ∼100 fs pulses were spectrally filtered using a diffractive 4f pulse shaper. The beam polarisation was controlled using a UV Glan polariser and half-wave plate. The angle of the incident light was controlled to match the guided mode on the grating coupler by translating the incident beam across the Fourier plane of the objective. The incident laser spot size on the input grating was 6.5 µm. The incident laser angle, polarisation, and position relative to the input coupler edge were optimised by maximising the observed transmitted intensity at the output coupler for low incident powers.

The light coming out of the waveguide was collected by the same microscope objective used for excitation and sent to the detection apparatus by means of a non-polarising beamsplitter cube. The light was imaged onto the entrance slit of an imaging spectrometer using a pair of relay lenses and recorded on a thermo-electrically cooled CCD camera.

The laser powers used for the experiments were obtained by measurement using a commercial laser power meter. For low laser powers at the sample surface, below the sensitivity limit of the power meter, the power was measured at a position in the beam path before the laser beam was attenuated using commercial pre-calibrated UV-fused-silica reflective metallic neutral density (ND) filters (FRQ series from Newport/MKS). We confirmed that the attenuation factor of the ND filters was equal to that specified in the calibration data provided by the manufacturer. Thus we could measure the beam power at a position where it was well above the sensitivity limit of the power meter while having accurate knowledge of the much lower power at the sample surface.

Supplementary Discussion 5. Numerical Simulations

The manifestation of strong nonlinear effects, in particular spectral broadening, is also observed in direct numerical simulations. The dynamics of the photons and the excitons is described by a simple model of coupled equations for the slow varying amplitude of the photonic mode U and the excitonic mode ψ. Here we integrate these equations using the well known Split-Step method which has proven to be simple and efficient for the simulation of equations of this kind. The method is described in supplementary reference 6, Chapter 7. See also supplementary reference 7 where this approach is used to describe the propagation of nonlinear pulses in GaAs waveguides. In that reference a non-dispersive photon mode was used and only the exciton blueshift mechanism for the nonlinearity was considered. In this work we use a very similar model but take into account the dispersion of the photon mode and both the exciton blueshift and Rabi quenching mechanisms for the nonlinearity (see the main text or Supplementary Discussion 8 for details of this terminology). We assume that the vertical structure (along the z coordinate) of the mode is fixed and the dispersion of the guided modes is known from fitting of the experimental polariton dispersion using the coupled oscillator model and photonic dispersion calculated from a solution of Maxwell's equations (see Supplementary Discussions 1 and 3). Let us mention that in spatially uniform systems the frequency of a plane wave depends only on the absolute value of the wavevector and thus the dispersion $\omega(k_x, k_y)$ reduces to $\omega(k)$ with $k = \sqrt{k_x^2 + k_y^2}$. In what follows we consider wave envelopes of finite duration and aperture propagating along the x coordinate. Assuming that the duration and the aperture are large (so that in the spectral domain the pulse is narrow) we can look for a solution in the form $E = U(t, x, y)\exp(i\omega_0 - ik_0x)$ for the electric field and $\Psi = \psi(t, x, y)\exp(i\omega_0 - ik_0x)$ for the exciton field. The frequency ω_0 is the frequency of the exciton resonance and k_0 is the wavevector of the uncoupled photon mode at which it crosses ω_0, such that $\omega_0 = \omega(k_0)$. Then for the slow varying amplitudes $U(t, x, y)$ and $\psi(t, x, y)$ we can write the system of coupled equations

$$\partial_t U = \hat{\omega} U - \gamma_p U + i\frac{\Omega}{1 + \beta|\psi|^2}\psi + p(t, x, y), \qquad \text{(S1a)}$$

$$\partial_t \psi = i\alpha|\psi|^2\psi - \gamma_x\psi + i\frac{\Omega}{1 + \beta|\psi|^2}U \qquad \text{(S1b)}$$

where $\hat{\omega}$ is the operator accounting for the photon subsystem dispersion and diffraction in the slow varying amplitudes representation, γ_p and γ_x account for the losses in the photon and in the exciton subsystems respectively, and Ω is the Rabi splitting in the linear regime. We assume that both the exciton frequency and the Rabi splitting depend on the exciton density $|\psi|^2$. This is accounted for by the Kerr like term in (S1b) (exciton blueshift mechanism) and by the dependence of the photon-exciton interaction strength $\frac{\Omega}{1+\beta|\psi|^2}$ on the exciton density (Rabi quenching mechanism). The parameters α and β control the strengths of the exciton blueshift and Rabi quenching nonlinear mechanisms respectively. In the Fourier representation the operator $\hat{\omega}$ can be approximated by a Taylor expansion in the vicinity of the point $k_x = k_0$, $k_y = 0$. Then the polynomial approximation of the operator can be written as $\omega(k_x, k_y) = \sum_n \sum_m \frac{1}{n!m!} \partial^m_{k_x} \partial^n_{k_y} \omega|_{k_x=k_0,\, k_y=0} q_x^m k_y^n$, where $q_x = k_x - k_0$. The approximation of the dependency $\omega(k)$ is known from a combination of a full electromagnetic calculation of the waveguide photonic modes and fitting to the experimental data, which allows us to calculate the coefficients in the expansion. We note that the essential criteria for reproducing the experiment is that the *polariton* rather than the photon dispersion is accurately represented. This is achieved by fitting the experimental polariton dispersion using the coupled photon and exciton equations in the linear regime with the photon dispersion taken from the electromagnetic calculation. Details are given in Supplementary Discussions 1 and 3.

In coordinate representation the operator $\hat{\omega}$ has the form $\hat{\omega} = \sum_n \sum_m \frac{1}{n!m!} \partial^m_{k_x} \partial^n_{k_y} \omega|_{k_x=k_0,\, k_y=0} (i\partial_x)^m (i\partial_y)^n$. Let us mention that if the dispersion of the photon mode is neglected and so $\omega = v_g k$ then, accounting only for the terms up to second order, we obtain $\hat{\omega} = v_g(\partial_x + \frac{1}{2k_0}\partial_y^2)$. In this simple case the model coincides exactly with the model used in supplementary reference 7. In this work we take into account the real photon dispersion and we use the time as an evolution coordinate. If the problem is posed in this way then the initial condition is the spatial distribution of the field. It is also possible to account for the excitation pulse by a source p in (S1b). Then we can set the zero initial condition and excite the field by the source to reproduce the effect of the incident pulse in the experiment. The pulse duration and the frequency detuning of the pulse from the linear exciton resonance is taken into account by the temporal dependency of the source p. The spatial dependency of the source p accounts for the spatial distribution of the incident pulse and the structure of the coupler. For the numerical simulations the parameters were tuned to fit the experiment at temperature $T = 100$ K and pump wavelength $\lambda = 354$ nm.

The evolution of the pulse as it propagates is shown in Supplementary Fig. 3 for two different pulse energies. From microscopic considerations (see Supplementary Discussion 8) it is expected that the Rabi quenching mechanism has a larger contribution than the exciton blueshift in this system so the simulations presented here are initially done for the case $\alpha = 0$. We later show that both types of nonlinearity give qualitatively the same behaviour. It is seen that during propagation in the nonlinear regime the pulse becomes slightly broader in the y direction but gets significantly compressed in the x direction because of the interplay of the normal dispersion, diffraction and effective defocusing nonlinearity. Figs. 3b,e correspond to the experimental device length of 100 µm. Even at pulse energy $W_0 = 200$ pJ the pulse has compressed and become modulated over the length of the device. At $W_0 = 750$ pJ the pulse is strongly modulated even by 50 µm propagation (Fig. 3d). After the initial compression and modulation the pulse begins to spread out due to dispersion and diffraction as can be seen by comparing the intensity envelopes for 150 µm (Figs. 3c and 3f) with those at 100 µm (Fig. 3b and 3e). This is because system losses reduce the total intensity so that, at longer propagation distances, the nonlinearity cannot compensate the dispersion.

We assume that light is rapidly coupled out by the output coupler over a length scale small compared to the coupler size. In this case the size of the output coupler is not important. This is justified by the experimental measurment of 3.5 µm decay length on the coupler, compared to 100 µm coupler size. To reproduce the experimental conditions the output coupler in numerical simulations is situated on the axis of the radiation propagation ($y_0 = 0$) at the distance of x_0 from the excitation spot. The calculated time-dependencies of the normalized field intensity $|U|^2$ at $x = x_0$, $y = 0$ are shown in Supplementary Fig. 4a for different energies of the initial pulse. It is seen that for higher pulse energies the compression of the pulse takes place. It is worth mentioning here that the exciton blueshift mechanism can lead to a similar effect. The field intensity distributions calculated for the case of exciton blueshift mechanism only are shown in Supplementary Fig. 4b. It is seen that the compression of the pulse is very much similar to the one observed for the case of Rabi quenching only (Supplementary Fig. 4a).

Now let us discuss the spectra of the output field. We define the temporal spectrum of the field measured at point x_0 as $S(\omega) = \int |\int U(x = x_0, y, t) \exp(-i\omega t)dt|^2 dy$. This quantity is equivalent to the experimental value obtained by the integration of the spectra measured at all points across the coupler. The spectra calculated for the incident pulse energies $W_0 = 115$ pJ and $W_0 = 750$ pJ are shown in Supplementary Figs. 5a,b for the case where the exciton-photon coupling depends on the exciton density. For relatively low intensities the calculated dependencies of the pulse spectral width match the experimental one well. At the same time the comparison shows that for higher intensities the model no longer has quantitative agreement with the experiment. Possible sources for this disagreement are discussed in the manuscript and encompass various aspects of semiconductor physics not captured by the model as well as gradual build-up of numerical error due to slight departure from the strict

validity of the slowly varying envelope approximation.

It is instructive to show the dependencies of the pulse spectral width on the energy in the incident pulse. We define the spectral width as $\Delta S = \lambda_1 - \lambda_2$, where $\lambda_2 = \frac{2\pi c}{\omega_2+\omega_0}$, $\lambda_1 = \frac{2\pi c}{\omega_1+\omega_0}$ and $\omega_{1,2}$ are found from the condition $\int_{-\infty}^{\omega_1} S(\omega)d\omega = \frac{1}{4}W$, $\int_{\omega_2}^{\infty} S(\omega)d\omega = \frac{1}{4}W$, $W = \int_{-\infty}^{\infty} S(\omega)d\omega$. Physically it means that a quarter of pulse energy is in the frequencies higher than ω_2 and a quarter of the pulse energy is in the frequencies lower then ω_1. The theoretical and experimental dependencies of the spectrum width are shown in Supplementary Fig. **5**c. It is seen that for relatively low pulse energies the numerical dependency matches the experimental one well, however at higher energy levels the deviation increases. This can be seen in more detail in Supplementary Fig. **5**d which shows the experimental and theoretical dependencies over a large range of pulse energies.

As already mentioned above, the effective defocusing nonlinearity has two contributions - from exciton blueshift and from Rabi quenching. We have modelled the spectral broadening for both types of nonlinearity. We find that the nonlinearity arising from the exciton blueshift does not lead to any qualitative differences in the spectral broadening of the pulse compared to that arising from Rabi quenching. This can be seen in Supplementary Fig. **5**d where the dependencies of the spectral width on the pulse energy are presented for both types of nonlinearity on their own as well as for the case where they contribute equally to the spectrum broadening. It is seen that the curves are very much similar. However, it is good to note that the nonlinear Rabi splitting gives slightly better fit to the experimental dependency, which can be seen as an indication that this is the dominating source of the nonlinearity. This is consistent with the calculation of the relative strengths taken from microscopic considerations (Supplementary Discussion 8).

Supplementary Discussion 6. Self phase modulation fitting and deduction of nonlinear refractive index

A light pulse travelling through a medium with Kerr-like nonlinearity accumulates a nonlinear phase proportional to its time-varying intensity envelope and the distance travelled. The time-varying phase then corresponds to new spectral components. This process is known as self-phase-modulation (SPM). We refer the reader to supplementary reference 8 or to other text books on nonlinear optics for a detailed discussion of SPM and derivation of the main results which we will use here. The accumulated nonlinear phase at the peak of the pulse can be written:

$$\delta\phi = \frac{2\pi}{\lambda_0} n_2 \frac{P_{peak}}{A_{\text{eff}}} L_{\text{eff}} \tag{S2}$$

where n_2 is the effective nonlinear refractive index which we are trying to find, $\lambda_0 = 353.4$ nm is the wavelength at the center of the pulse spectrum, $A_{\text{eff}} = 1\,\mu\text{m}^2$ is the waveguide effective nonlinear cross-sectional area[8], $P_{\text{peak}} = E_{\text{pulse}}/T_0$ is the laser pulse peak power obtained from the pulse energy E_{peak} and temporal width T_0, and $L_{\text{eff}} = L_{\text{loss}}\left[1 - \exp\left(-L/L_{\text{loss}}\right)\right]$ is the effective distance over which the nonlinearity acts accounting for loss. The device length is L and L_{loss} is the characteristic decay length due to loss. In calculating A_{eff} we have used the 79 nm FWHM waveguide mode profile obtained from Lumerical FDTD simulations (see Supplementary Discussion 7) for the intensity profile in the z direction (see Fig. 1 in the main text) and the 6.5 µm FWHM Gaussian spot at the input as the transverse (y) intensity profile, noting that the spot did not diffract significantly over the 100 µm device length at the low powers where we perform this fitting. We used the standard nonlinear optics formula[8] in calculating A_{eff}. It essentially comes from a cross-sectional average of the nonlinear change in refractive index weighted by the intensity of the mode.

To quantify the intensity-dependent spectral width at low powers we fit the data shown in Fig. 5a,b in the main text with SPM broadened spectra corresponding to different peak nonlinear phases $\delta\phi$. Before describing the fitting we briefly comment on the validity of the model. From the measured polariton dispersion relation (see Supplementary Discussion 3) we obtain the second order dispersion coefficient $\beta_2 < 250$ ps^2 m^{-1}. Combined with the spectral FWHM $\hbar\Delta\omega < 5$ meV the characteristic dispersion length[8] $L_D = 4\log(2)/\left(\beta_2\Delta\omega^2\right) >$ 200 µm, which is much larger than the loss length and the device length, so that dispersion can be neglected. For low powers, where the pulses accumulate small nonlinear phase, only the leading (third order) term in the system nonlinear response is significant so that the waveguide can be described as an effective Kerr-like waveguide with nonlinear refractive index n_2. We note that in the polariton system both the exciton blueshift and Rabi quenching effects are third order nonlinear processes to leading order. In this regime of low power and negligible dispersion the model of SPM may be applied and used to find n_2. We note that at the higher pulse energies shown in Fig. 5 in the main text the experimental spectra become slightly asymmetric, which arises due to the frequency dependent polariton nonlinearity. This effect cannot be captured by a simple SPM model, but least squares fitting averages over the spectral components on either side of the peak to give an average value of the phase. The phases obtained for these higher pulse energies lie on a straight line with those at lower pulse energy (Fig. 5c,d in the main text) where the asymmetry is negligible, showing that this approach works well.

For each pulse energy we deduce the nonlinear phase at the peak of the pulse by fitting the experimental spectrum with modelled SPM-broadened spectra. The experimental spectra and fits are shown in Figs. 5a,b in the main text on a logarithmic scale. At the lowest powers, SPM manifests as a growth of low intensity spectral side-bands which eventually become comparable to the

main peak as the power is increased[8]. We therefore fit the model to the logarithm of the spectra (as shown in the figure) since this maximises the sensitivity to these low intensity side-bands. The fit was performed using experimental points down to -20dB where the data is at least a factor of 2 above the noise floor. The initial (zero power limit) pulse used in the SPM model was the unchirped Gaussian pulse whose spectrum was the best fit for the experimental one in the linear regime, having temporal FWHM $T_0 = 425 \pm 4$ fs. Using an unchirped Gaussian pulse shape is equivalent to assuming a maximum pulse peak power for a given pulse energy. If the pulse was chirped or had a different shape then the peak power would be lower and the actual values of n_2 would be larger than those we deduce.

We plot the peak phase vs. pulse energy obtained from the fitting in Figs. 5c,d in the main text for two temperatures along with the best fit straight lines. The phase has a linear dependence on pulse energy as expected for an SPM spectral broadening mechanism, which confirms the validity of this model. The linear fitting gives a slope of 50 ± 10 pJ rad^{-1} for 100 K and 32 ± 9 pJ rad^{-1} for 200 K. From this, n_2 can be obtained using Eqn. (S2). The effective nonlinear length is given by $L_{\rm eff} = L_{\rm loss}(1 - \exp(L/L_{\rm loss}))$ where $L_{\rm loss}$ is the decay length (see Supplementary Discussion 7) and $L = 100\,\mu$m is the device length. We find $n_2 = (1.9 \pm 0.3)\times 10^{-13}\,{\rm cm}^2\,{\rm W}^{-1}$ for 100 K and $n_2 = (3.7 \pm 1.0)\times 10^{-13}\,{\rm cm}^2\,{\rm W}^{-1}$ for 200 K. The difference between the values at different temperatures arises partly because of the different losses but mainly because the pulse central frequency is closer to the exciton resonance at 200 K so that the polaritons have a higher exciton fraction and are expected to exhibit a larger nonlinearity (see Supplementary Discussion 8).

We finally comment on how we obtain the uncertainties we give for n_2. They were obtained using the uncertainties in $\delta\phi$, T_0, $E_{\rm pulse}$, and $L_{\rm eff}$, where the latter comes from $L_{\rm loss}$. The uncertainties in $\delta\phi$ and T_0 come from the least squares fitting procedure (see above). The uncertainties in $E_{\rm pulse}$ and $L_{\rm loss}$ come from a combination of the FDTD calculation of the input coupling efficiency $(\kappa_{\rm up}/\kappa)\,F$ and the experimental measurement of the output power vs. incident power η (see Supplementary Discussion 7). It is important to note that $L_{\rm loss}$ and the coupling efficiency are not independent quantities. Our measurement of η fixes a relationship between them through Eqn. (S3). This has the effect of substantially reducing the uncertainty in the final values of n_2, which may be understood qualitatively in the following way. If the coupling efficiency was at the higher end of the confidence interval we obtained using our FDTD simulations then, for a given *incident* pulse energy, the coupled pulse energy $E_{\rm pulse}$ would be higher. However, because η is a fixed measured value, a higher coupling efficiency also implies higher losses (Eqn. (S3)) so that the length $L_{\rm eff}$ over which the nonlinear phase builds up would be shorter. This shorter $L_{\rm eff}$ partially cancels out the higher $E_{\rm pulse}$ so that there is not a strong change in the value of n_2 needed to obtain the measured $\delta\phi$. Thus the deduced n_2 is not highly sensitive to the FDTD value of coupling efficiency - the dependence is partially cancelled out because η has been measured. Quantitatively, there is some uncertainty in the measurement of η itself. To rigorously account for the uncertainties in all these co-variant quantities we propagate the errors using a Monte-Carlo calculation where the independently determined quantities (η, $\delta\phi$, T_0, coupling efficiency) are treated as normally distributed random variables with standard deviation corresponding to their measured uncertainties. We then sample from these distributions and calculate n_2 using Eqn. (S2), repeating the process many times. We are left with a histogram of n_2 values with a standard deviation from which we obtain the uncertainty.

Supplementary Discussion 7. Coupling efficiency and losses

We determine the coupling efficiency and propagation losses using a combination of measurements and electromagnetic modelling (finite-difference time-domain method, FDTD). Here we explain our method by way of an example using the data presented in Fig. 5 in the main text.

We first measured the ratio of power coming out from the output coupler vs. that incident on the input coupler. The incident power was measured directly using a power meter. The power coming out from the output coupler was below the sensitivity of the power meter and so it was measured using the count rate on the CCD camera. The CCD count rate vs. laser power at the sample surface was calibrated by sending the excitation laser at a known power to the CCD along exactly the same path used for detection. For this calibration the laser power was measured with the power meter before the beam was heavily attenuated with neutral density filters (see Supplementary Discussion 4). We obtained the ratio output power divided by incident power $\eta = (4.3 \pm 0.4) \times 10^{-5}$ and $(1.8 \pm 0.2) \times 10^{-5}$ for temperatures 100 K and 200 K respectively.

The output vs. incident power ratio η arises from the input and output coupling efficiency and the propagation losses and can be expressed as

$$\eta = (\kappa_{\rm up}/\kappa)^2 \cdot F \cdot \exp\left(-L/L_{\rm loss}\right). \tag{S3}$$

The lengths L and $L_{\rm loss}$ are, respectively, device length and the characteristic propagation loss length. The rate $\kappa_{\rm up}$ is the coupling rate of the guided mode to the free space propagating mode above the sample used for excitation/detection while κ gives the coupling of the guided mode to all channels including to the excitation/detection mode and into the substrate. It also includes Ohmic losses in the metallic coupler. The ratio $\kappa_{\rm up}/\kappa$ is squared since, by reciprocity, this factor is the same for the input and output couplers. The factor F accounts for the imperfect mode matching of the Gaussian

excitation beam to the exponentially decaying guided mode at the input coupler. The highest possible value of F for a Gaussian shaped beam is 0.8, which occurs when the spot size is matched to the decay length of the mode on the grating and the spot is positioned optimally with respect to the grating edge (see e.g. the discussion in the supplementary material of supplementary reference 9). In our experiment the spot size and decay length were comparable at 6.5 μm and 3.5 ± 0.4 μm respectively and in each experiment we carefully optimised the spot position and incidence angle (see Supplementary Discussion 4). We therefore assume optimal coupling, $F = 0.8$, and note that if the real value is smaller then less power would be coupled into the waveguide than we assume so that the value of n_2 would be larger than we deduce.

To evaluate $\kappa_{\mathrm{up}}/\kappa$ we performed FDTD simulations of the waveguide and grating structure using the commercial Lumerical FDTD package. We used the full 3D solver but, since our structure is homogeneous in the y direction (transverse to the propagation) we assumed plane-wave behaviour in the y direction to reduce simulation run times. This is well justified for the non-diffracting spot sizes we have in the experiment in the low power regime where we calibrate the coupling efficiencies. The guided mode of the (simulated) waveguide was excited away from the output coupler and the fraction of power in the guided mode which is coupled into the free space above the output grating was recorded. The nitride material refractive indexes are the same as those used in Supplementary Discussion 1. The exact values of complex permittivity chosen for the nickel grating coupler were found to make some difference to the results so we performed the simulation using a range of experimental values from the literature[10–12] and took an average. We obtain $\kappa_{\mathrm{up}}/\kappa = (6 \pm 1)\%$ and $(5 \pm 1)\%$ for 200 K and 100 K temperature, respectively, where the uncertainties include the spread due to variation in material parameters between literature sources. The corresponding *input* coupling efficiencies are $F \cdot (\kappa_{\mathrm{up}}/\kappa) = 5 \pm 1\%$ and 4.2 ± 0.9 % for 200 K and 100 K, respectively. To check for consistency, the simulated decay length on the grating was 4.9 ± 0.2 (4.6 ± 0.2) μm for 200 K (100 K) which agrees well with the experimental value suggesting that the effect of the grating is accurately modelled. The simulated emission angle of the out-coupled light also agrees well with the experimentally measured values.

Taking these values of F, $\kappa_{\mathrm{up}}/\kappa$ and the experimentally measured values of η we are then able to deduce the losses in the waveguide. We obtain characteristic loss lengths $L_{\mathrm{loss}} = 25 \pm 3$ μm for 100 K and 21 ± 2 μm for 200 K. These are slightly smaller than the values $\sim 50 \pm 15$ μm reported for similar exciton fractions in supplementary reference 1 which were, however, measured between 4 K and 30 K. This difference could arise from the increased homogeneous exciton linewidth at elevated temperatures. These can even exceed the inhomogeneous broadening and begin to dominate below-band-gap absorption[1]. The increase in propagation losses between 100 K and 200 K may be due to a reduction in internal quantum efficiency of the multi-quantum wells, which was previously observed in photoluminescence measurements on the near-identical sample of supplementary reference 1. It originates due to increased scattering of excitons away from the radiatively coupled states at higher temperatures[3].

We finally note that the uncertainties in all the parameters we discuss in this section are robustly incorporated into the uncertainty we give for the nonlinear parameter n_2 (see Supplementary Discussion 6).

Supplementary Discussion 8. Estimate of nonlinear refractive index from first-principles calculations

The nonlinearity in our polariton waveguide is due to a combination of two microscopic mechanisms[13]. Increasing intensity causes an increase (blueshift) of the exciton frequency by an amount ΔE_{X}. We refer to this as the exciton blueshift mechanism. Increasing intensity also reduces the exciton oscillator strength resulting in a change in Rabi splitting, ΔE_{sat}. We refer to this as the Rabi quenching mechanism. Both of these effects lead to a frequency blueshift of the lower polariton branch for a given wavenumber or, equivalently, a decrease in wavenumber for any given frequency. This nonlinear change in wavenumber vs. frequency is equivalent to a (frequency dependent) nonlinear change in refractive index. We will first consider the size of the excitonic blueshift and Rabi quenching parameters following the method given in supplementary reference 13 and using the GaN QW exciton Bohr radius[14]. We will then express the nonlinear response as a nonlinear refractive index.

The blueshift and change in Rabi splitting are, to first order,

$$\Delta E_{\mathrm{X}} = g_{\mathrm{XX}} n_{\mathrm{X}}, \tag{S4}$$

$$\Delta E_{\mathrm{sat}} = \beta_{\mathrm{X}} n_{\mathrm{X}}, \tag{S5}$$

where g_{XX} and β_{X} are interaction constants and n_{X} is the number of excitons per unit area per quantum well (exciton density). Note that β_{X} is negative since the Rabi splitting decreases with increasing exciton density.

The exciton blueshift interaction constant is given by[13]:

$$g_{\mathrm{XX}} = \frac{3e^2 a_{\mathrm{B}}}{4\pi\epsilon_0\epsilon}, \tag{S6}$$

where e is the electron charge, a_{B} is the exciton effective Bohr radius, $\epsilon \sim 10.28$ is the static dielectric constant[15], and ϵ_0 is the vacuum permittivity. The exciton effective Bohr radius can be expressed as:

$$a_{\mathrm{B}} = a_{\mathrm{B}}^{3D}\left(1 + \frac{d-3}{2}\right)^2, \tag{S7}$$

where $a_{\mathrm{B}}^{3D} \sim 3.2$ nm is the bulk exciton Bohr Radius and d is the fractional dimensionality of the excitonic system[14]. The fractional dimensionality is in turn given by:

$$d = 1 + 2\sqrt{\frac{Ry^*}{E_{\mathrm{X}}^{B}}}, \tag{S8}$$

where $Ry^* = 25$ meV is the bulk exciton Rydberg[16], and $E_{\mathrm{X}}^{B} = 40$ meV is the exciton binding energy[1], which in our case yields a fractional dimensionality of $d = 2.58$. This then gives an effective exciton Bohr radius of $a_{\mathrm{B}} = 2.0$ nm.

The interaction constant for Rabi quenching is given by[13]:

$$\beta_{\mathrm{X}} = -\frac{8\pi}{7}\Omega a_{\mathrm{B}}^2, \tag{S9}$$

where $\Omega \sim 90$ meV is the Rabi splitting.

We finally obtain the exciton blueshift per unit density per quantum well $g_{\mathrm{XX}} \sim 0.85\ \mu\mathrm{eV}\ \mu\mathrm{m}^2$ and the change in Rabi splitting per unit density per quantum well is $\beta_{\mathrm{X}} \sim$ -1.3 $\mu\mathrm{eV}\ \mu\mathrm{m}^2$.

When one considers the total system of waveguide photonic mode coupled to $N = 22$ QWs, the theoretical value of the effective nonlinear refractive index, n_2, may be derived from the first order expansion of the change in wavenumber vs. frequency of the polariton dispersion. It is given by:

$$|n_2| = \frac{n_g^2 H}{c_0(\hbar\omega)^2}\frac{|X|^4}{|C|^4}\left(\frac{g_{\mathrm{XX}}}{N} - \frac{\beta_{\mathrm{X}}}{N}\frac{|C|}{|X|}\right), \tag{S10}$$

where $n_g = 4.9$ (5.4) is the uncoupled-photon group velocity index at 100 K (200 K) (with the frequency dependence coming from the nitride material dispersion), $H = 79$ nm is the guided mode width (FWHM) in the z direction obtained from FDTD calculation, c_0 is the light speed in vacuum, $\hbar\omega = 3.51$ eV is the frequency of the pulse in energy units, and $|X|^2/|C|^2 = 0.31 \pm 0.05$ (0.42 ± 0.06) is the ratio of excitonic and photonic fractions of the polaritons at 100 K (200 K). From these, we obtain the theoretical values of $n_2 = (3.3 \pm 0.9) \times 10^{-13}\,\mathrm{cm}^2\,\mathrm{W}^{-1}$ and $(6.5 \pm 1.5) \times 10^{-13}\,\mathrm{cm}^2\,\mathrm{W}^{-1}$ at $T = 100$ K and 200 K, respectively.

We finally comment on the relative contributions of the exciton blueshift and Rabi quenching mechanisms to n_2. As can be seen from Eqn. (S10) the ratio of Rabi quenching to exciton blueshift contributions is $(\beta_{\mathrm{X}}|C|)\,/\,(g_{\mathrm{XX}}|X|)$. For 100 K and 200 K respectively we obtain ratios 2.75 and 2.36 respectively. Thus we expect that the contributions are similar but that the Rabi quenching provides the larger contribution.

* p.m.walker@sheffield.ac.uk
† raphael.butte@epfl.ch

[1] Ciers, J. *et al.* Propagating polaritons in III-nitride slab waveguides. *Phys. Rev. Appl.* **7**, 034019 (2017).
[2] Bienstman, P. & Baets, R. Optical modelling of photonic crystals and VCSELs using eigenmode expansion and perfectly matched layers. *Optical and Quantum Electronics* **33**, 327–341 (2001).
[3] Ciers, J. A. S. *Light-Matter Interaction in III-Nitride Waveguides: Propagating Polaritons and Optical Gain.* Ph.D. thesis, Institute of Physics, EPFL, Lausanne (2018).
[4] Brunner, D. *et al.* Optical constants of epitaxial AlGaN films and their temperature dependence. *J. Appl. Phys.* **82**, 5090–5096 (1997).
[5] Carlin, J.-F. *et al.* Progresses in III-nitride distributed bragg reflectors and microcavities using AlInN/GaN materials. *Phys. Stat. Sol. (b)* **242**, 2326–2344 (2005).
[6] Yang, J. *Nonlinear Waves in Integrable and Nonintegrable Systems* (Society for Industrial and Applied Mathematics, Philadelphia, PA, 2010).
[7] Walker, P. M. *et al.* Ultra-low-power hybrid light–matter solitons. *Nat. Commun.* **6**, 8317 (2015).
[8] Agrawal, G. P. *Nonlinear Fiber Optics* (Academic Press, San Diego, 2001), 3rd edn.
[9] Walker, P. M. *et al.* Dark solitons in high velocity waveguide polariton fluids. *Phys. Rev. Lett.* **119**, 097403 (2017).
[10] Johnson, P. & Christy, R. Optical constants of transition metals: Ti, V, Cr, Mn, Fe, Co, Ni, and Pd. *Physical Review B* **9**, 5056–5070 (1974).
[11] Werner, W. S. M., Glantschnig, K. & Ambrosch-Draxl, C. Optical constants and inelastic electron-scattering data for 17 elemental metals. *Journal of Physical and Chemical Reference Data* **38**, 1013–1092 (2009).
[12] Lynch, D. W. & Hunter, W. Comments on the optical constants of metals and an introduction to the data for several metals. In Palik, E. D. (ed.) *Handbook of Optical Constants of Solids*, 275–367 (Academic Press, Orlando, Fla., 1997).
[13] Brichkin, A. S. *et al.* Effect of Coulomb interaction on exciton-polariton condensates in GaAs pillar microcavities. *Phys. Rev. B* **84**, 195301 (2011).
[14] Christmann, G. *et al.* Large vacuum Rabi splitting in a multiple quantum well GaN-based microcavity in the strong-coupling regime. *Phys. Rev. B* **77**, 085310 (2008).
[15] Bernardini, F., Fiorentini, V. & Vanderbilt, D. Polarization-based calculation of the dielectric tensor of polar crystals. *Phys. Rev. Lett.* **79**, 3958–3961 (1997).
[16] Kornitzer, K. *et al.* Photoluminescence and reflectance spectroscopy of excitonic transitions in high-quality homoepitaxial GaN films. *Phys. Rev. B* **60**, 1471–1473 (1999).

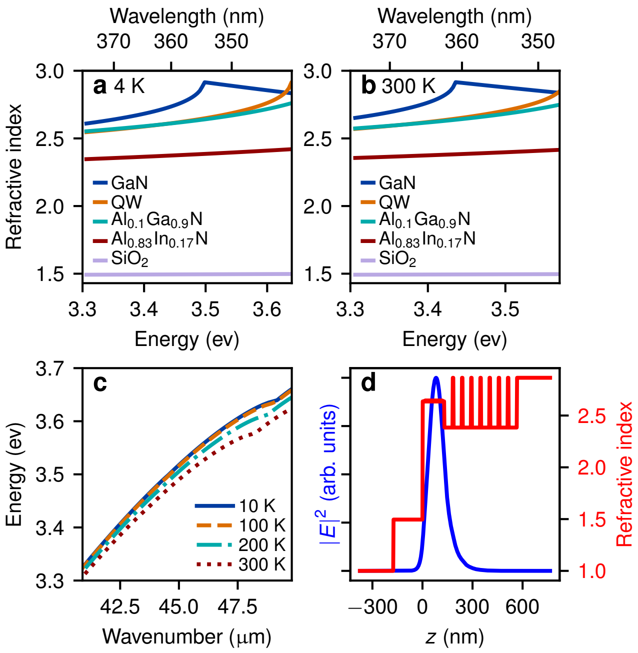

Supplementary Figure 1. **Waveguide photonic modes** **a** Refractive indexes of the waveguide materials at 4 Kelvin. **b** Refractive indexes of the waveguide materials at 300 Kelvin. **c** Dispersion relations of the waveguide photon modes in the absence of strong photon-exciton coupling for several temperatures. **d** Guided mode electric field intensity and refractive index as a function of position along the growth direction z (refer to Fig. 1a in the main text for coordinate axes). Calculated for 4 Kelvin and wavelength 355 nm (3.493 eV). Position $z = 0$ corresponds to the interface between the AlInGaN waveguide core and SiO_2 upper cladding.

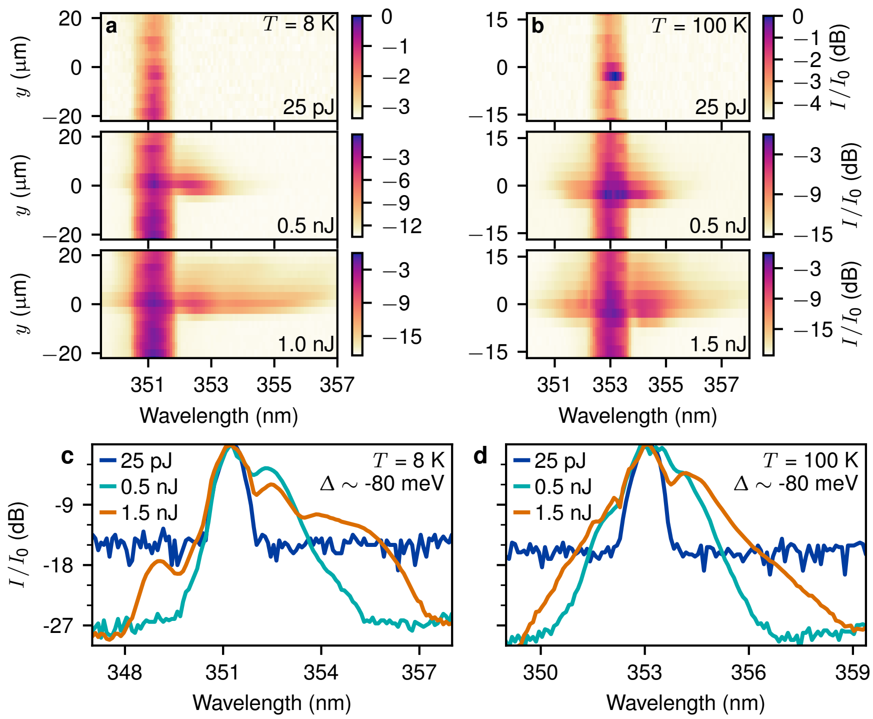

Supplementary Figure 2. **Experimental spectra after nonlinear pulse propagation. (a,b)** Colour maps of the output intensity vs. wavelength, λ, and position y transverse to the propagation direction for increasing pulse energy coupled into the waveguide and at temperatures $T = 8$ K (**a**) and at $T = 100$ K (**b**). The injected pulses had central wavelength $\lambda = 351$ nm (**a**) and 353 nm (**b**) and propagation distance $L = 100$ μm. **(c-d)** Spectra integrated along y for $T = 8$ K (**c**) and 100 K (**d**), both at pulse-exciton detuning $\Delta \sim$ -80 meV.

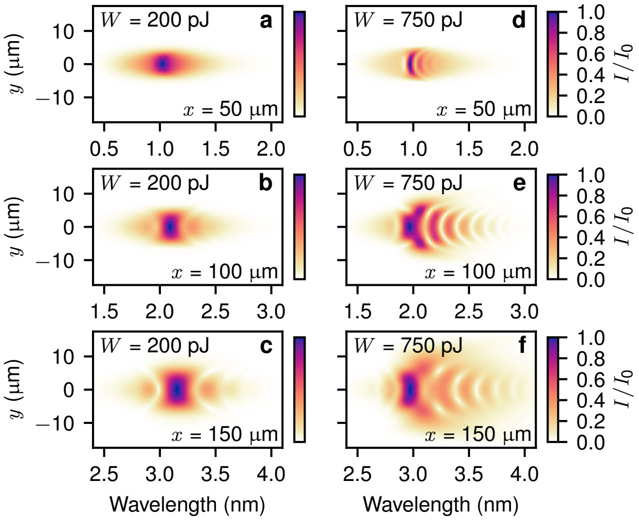

Supplementary Figure 3. **Calculated spatio-temporal distributions of the intensity of the field propagating in the planar waveguide.** The left column (panels **a-c**) shows the nonlinear propagation with pulse energy $W_0 = 200$ pJ while the right column (panels **d-f**) shows the same for $W_0 = 750$ pJ. No significant change in pulse was observed in the linear propagation regime ($W_0 = 2.5$ pJ). Time $t = 0$ corresponds to the intensity peak of the incident pulse. Pulse parameters correspond to the experiment for central wavelength $\lambda = 354$ nm.

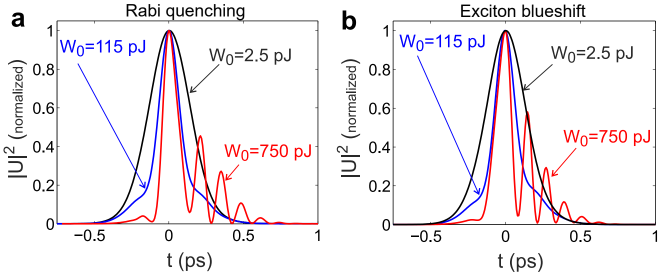

Supplementary Figure 4. **Calculated temporal intensity profiles at the transverse centre of the output coupler ($y = 0$, $x = x_0 = 100\,\mu$m) for different pulse energies.** The panels compare the cases where the nonlinearity is caused by Rabi quenching (**a**) and by exciton blueshift (**b**). Curves are shifted so that the peak is at $t = 0$ and simulations are for pulse central wavelength $\lambda = 354$ nm.

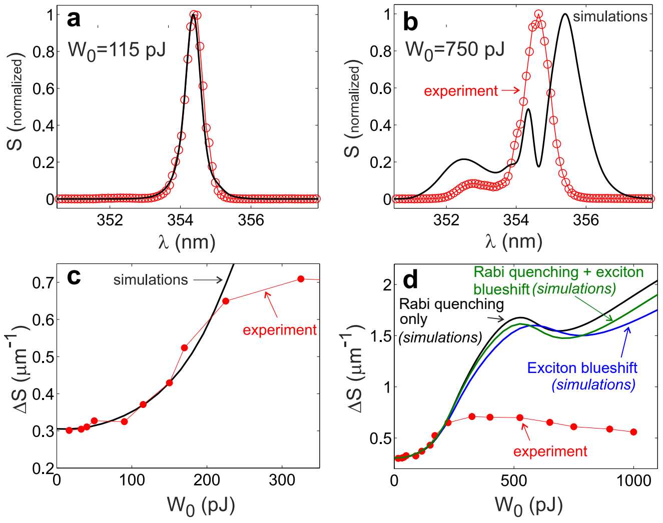

Supplementary Figure 5. **Comparison of numerically calculated and experimental spectra at the output.** (**a**) and (**b**) compare numerical (black lines) and experimental (open red circles) spectra for incident pulse energies W = 115 pJ and W = 750 pJ respectively. (**c**) and (**d**) compare numerical and experimental dependence of the spectral width on pulse energy for small and large ranges of pulse energy respectively. The simulations for **(a-c)** are done for the case when the nonlinearity is caused solely by the Rabi quenching. (**d**) compares nonlinearities caused by the Rabi quenching (black curve), exciton blueshift (blue curve), and an equal contribution of both (green curve).
